# Clinicopathological correlation of Cathepsin K expression in salivary gland carcinomas; relation to patients` outcome

**DOI:** 10.1186/s13000-023-01353-5

**Published:** 2023-05-17

**Authors:** Heba Ahmed Elhendawy, Samar Soliman

**Affiliations:** Oral Pathology Department, Faculty of Dentistry, Mansoura University, Mansoura, Egypt

**Keywords:** Salivary gland carcinomas, Cathepsin K, Immunohistochemistry, Distant metastasis, DFS, OS

## Abstract

**Background:**

Salivary gland carcinomas (SGCs) represent various groups of tumors that demonstrate marked diversity in their prognosis owing to different histology and clinical characteristics. One of the poor prognostic indicators is distant metastasis which is considered the major reason for death in SGC patients. Discovering new biomarkers is urgently required to aid in the detection of cancer onset and progression. Cathepsin K (CTSK), the lysosomal cysteine protease has a principal role in cancer invasion and progression through interaction with the tumor microenvironment, degradation of extracellular membrane proteins and destruction of the elastic lamina of blood vessels. In the English literature, little information was present about the role of CTSK in SGCs. The current study aimed to assess the immunohistochemical expression of CTSK in SGCs and correlate its expression to different clinicopathologic parameters.

**Methods:**

The retrospective study applied to 45 cases of SGCs categorized as high-grade (33 cases) and low-grade SGCs (12 cases) following the criteria of WHO classification (2017) of head and neck tumors. All patients` clinicopathological and follow-up records were retrieved. The following statistical tests were used to study the variance of CTSK expression in SGCs concerning different clinicopathological parameters; Pearson`s Chi-square test, unpaired two-tailed student t-test, One-way ANOVA, and Post Hoc tests. Disease-free survival (DFS) and Overall survival (OS) were calculated and displayed with the Kaplan–Meier strategy and analyzed with the log-rank test. Univariate and multivariate survival analyses were performed with Cox regression. A *P*-value lesser than 0.05 was considered statistically significant.

**Results:**

Strong CTSK expression was significantly related to high-grade SGCs (*P* = 0.000), large infiltrating carcinomas (*P* = 0.000), presence of nodal (*P* = 0.041) and distant metastasis (*P* = 0.009), advanced TNM clinical stage (*P* = 0.000), the incidence of recurrence (*P* = 0.009), and reduced DFS (*P* = 0.006). Distant metastasis was the independent predictor for DFS using Cox regression model.

**Conclusions:**

CTSK has a great role in cancer progression by triggering many signaling pathways. Its level in cancerous tissue is considered an effective index for predicting the severity and prognosis of cancer. Therefore, we indicate its utility as a prognostic tool and therapeutic target for cancer treatment.

**Trial registration:**

Retrospectively registered.

## Background

Salivary gland carcinomas (SGCs) represent various groups of tumors with different clinical characteristics and morphological patterns making them difficult to classify, identify, and treat [1, 2]. Additionally, because of their unexpected prognosis, SGCs play a significant role in the field of oral and maxillofacial pathology [3, 4]. Mucoepidermoid carcinoma (MEC) and adenoid cystic carcinoma (AdCC) are the most common SGCs [4]. Malignant salivary gland tumors demonstrate marked diversity in their prognosis owing to different histologies, the age of the patient, and the status of metastasis and local invasion. Generally, children and teenagers often have a better prognosis than adults owing to the more distinct histology, the rarity of local invasion, and the lower rate of cervical metastasis [5, 6].

Among the poor prognostic indicators of survival, distant metastasis is considered the major reason of death for patients who are diagnosed with SGCs. Metastasis often involves the lungs, bone, liver, soft tissue, lymph nodes, and the brain [7–11]. Nodal and distant metastasis, high incidence of recurrence, and reduced survival often were reported in high-grade MEC, AdCC, and Carcinoma ex pleomorphic adenoma (CXPA). On the other hand, acinic cell carcinoma (ACC) showed better 5-year survival (75–96%), and lower incidence of cervical neck metastasis and distant metastasis [12–17].

Cancer is considered the second cause of death following ischemic heart disease, and by 2060 it will become the first [18]. There is an urgent need for discovering new biomarkers that aid in the early detection of cancer onset and progression [19, 20]. Cathepsin K (CTSK) is one of the lysosomal cysteine proteases and the most powerful collagenolytic endopeptidase. CTSK has high osteoclast expression and has an important role in bone resorption [21, 22]. CTSK has a principal role in cancer invasion and progression through interaction with the tumor microenvironment, degradation of extracellular membrane proteins, and destruction of the elastic lamina of blood vessels [23, 24]. Today, high CTSK expression has been reported in several neoplasms of epithelial and mesenchymal origin. Many pathways may be triggered in the mechanism by which CTSK could promote the proliferation, invasion, and migration of tumor cells (RANK/RANKL, TGF-B, mTOR, and Wnt/β-catenin pathways). Recently, the utility of CTSK inhibitors in cancer treatment reached some progress [25]. In the literature review, little information was present about the role of CTSK in salivary gland tumors. Therefore, the current study aimed to assess the immunohistochemical expression of CTSK in SGCs and correlate the expression to different clinicopathological parameters.

## Material and methods

### Patients’ selection and data retrieval

The present retrospective study worked on 45 SGCs that were selected from the archives of the Pathology laboratory and Oncology unit of the Oncology Center, Faculty of Medicine, Mansoura University. The study included 33 cases of high-grade SGC: (13 cases of high-grade MEC, 12 cases of AdCC, 8 cases of CXPA), and 12 cases of low-grade SGC (7 cases of low-grade MEC and 5 cases of acinic cell carcinoma (ACC) following the criteria of (2017) WHO classification of head and neck tumors [26]. Five blocks of normal salivary gland tissue that are present in the mucocele were used as a control group. Patients` clinicopathological and follow-up records were retrieved. All cases that were included in our study were primary SGCs that received surgical treatment and had follow-up records for three years. Cases with missed follow-up records or had small-sized tissue biopsies were excluded from the selection. The follow-up of the patients started after completion of the treatment by clinical examination and ultrasonography for the head and neck region, chest X-ray, bone scan, and abdominal ultrasonography were performed when relapse was suspected. Three years` overall survival (OS) and disease-free survival (DFS) data were obtained from the medical reports.

### Immunohistochemistry

The formalin fixed paraffin embedded tissue blocks were cut at 4 µm thickness. Tissue sections were placed on coated slides. Deparaffinization then rehydration in descending grades of alcohol followed by water. Antigen retrieval was performed with 0.01 M citric acid buffer (pH = 6.0) and heated for 10 min in a microwave. Then, sections were incubated in a blocking medium (3% H2O2) for 5 min followed by washing with distilled water. Anti CTSK (3F9, Abcam, 1:300) was used. Immunoreaction was performed using the streptavidin–biotin complex method and overnight incubation, the tissue sections were evaluated in a semiquantitative way assessing both staining intensity and percentage of positive cells as previously described [27–29]. The resulting score was calculated by multiplying the staining intensity (0 = no staining, 1 = mild staining, 2 = moderate staining, and 3 = strong staining) by the percentage of immunoreactive tumor cells (0 to 100). The immunostaining was considered 0 or negative when the score was < 25; 1 + or weak for score 26 to 100; 2 + or moderate for score 101 to 200; and 3 + or strong for score 201 to 300.

### Statistical analysis

The analysis of data was done by One-way ANOVA and Post hoc tests to study the variance of CTSK expression in SGCs concerning different clinicopathological parameters. The Chi-square test also was used for data analysis. Two-sided *P*-values were detailed for all investigations. Disease-free survival (DFS) and Overall survival (OS) were calculated and displayed with the Kaplan–Meier strategy and analyzed with the log-rank test. Univariate and multivariate survival analyses were performed with the Cox regression model to detect the independent prognostic factor. A *P*-value lesser than 0.05 was considered statistically significant. Statistical analysis of the data was done by using the Excel program and Statistical Package for Social Science (SPSS) version 22 program.

## Results

### Clinicopathological characteristics of the considered cases

As shown in Table 1, a total of 45 patients of SGCs were distinguished and involved in the study. Concerning gender, our work included 27 females (60%) and 18 males (40%), with female to male ratio of 1.5 to 1. The age range of the studied cases was from 35 to 90 years, with a mean of 65.69 years.

**Table 1:** Clinicopathologic characteristics of the worked cases of SGCs

**Clinicopathologic variables**		**frequency**	**%**
**Patient gender**	male	18	40.0
	female	27	60.0
**Tumor type**	Low-grade MEC	7	15.6
	High-grade MEC	13	28.9
	Adenoid cystic carcinoma	12	26.7
	Carcinoma ex pleomorphic adenoma	8	17.8
	Acinic cell carcinoma	5	11.1
**Histologic grade**	Low-grade carcinomas	12	26.7
	High-grade carcinomas	33	73.3
**Tumor site**	parotid salivary gland	23	51.1
	Submandibular salivary gland	12	26.7
	Soft and hard palate minor salivary glands	5	11.1
	Sublingual major salivary gland	4	8.9
	Retromolar mucosa	1	2.2
**Tumor size**	T1	7	15.6
	T2	12	26.7
	T3	17	37.8
	T4	9	20.0
**Status of nodal involvement**	positive nodal involvement	26	57.8
	negative nodal involvement	19	42.2
**Distant metastasis**	present	17	37.8
	absent	28	62.2
**TNM stage**	stage I	7	15.6
	stage II	6	13.3
	stage III	13	28.9
	stage IV	19	42.2
**Incidence of recurrence**	Present	17	37.8
	Absent	28	62.2
**Incidence of death**	Died	5	11.1
	Alive	40	88.9
**CTSK expression**	Negative expression	8	17.8
	Weak expression	8	17.8
	Moderate expression	6	13.3
	Strong expression	23	51.1
	**Total**	45	100

Frequency table

The study included 13 cases (28.9%) of high-grade MEC, 12 cases (26.7%) of AdCC, eight cases (17.8%) of CXPA, seven cases (15.6%) of low-grade MEC, and five cases (11.1%) of ACC. The parotid salivary gland was the most prevalent site of SGCs, more than one-half of the worked cases aroused from that gland (23 cases, 51.1%). The submandibular major salivary gland is the second site of involvement (12 cases, 26.7%), then minor salivary glands in the soft and hard palate (5 cases, 11.1%), followed by the sublingual major salivary gland (4 cases, 8.9%), and finally mucosa of the retromolar region (one case, 2.2%). Regarding tumor size, the greater number of the worked cases were large-sized carcinomas encountered in T3 and T4 (26 cases, 57.7%), while the remaining 19 cases (42.2%) were small-sized carcinomas (T1 and T2). According to the status of nodal and distant metastasis, 26 cases (57.8%) had positive tumor deposits in lymph nodes and 17 cases (37.8%) were reported to have distant metastasis to lung, brain, and bone that was confirmed by ultrasonography. During the three-years follow-up period, recurrence was reported in 17 cases (37.8%) and five cases (11.1%) died due to secondary complications associated with the disease.

### Cathepsin K (CTSK) immunohistochemical expression concerning the different clinicopathological variables

**Fig. 1:**
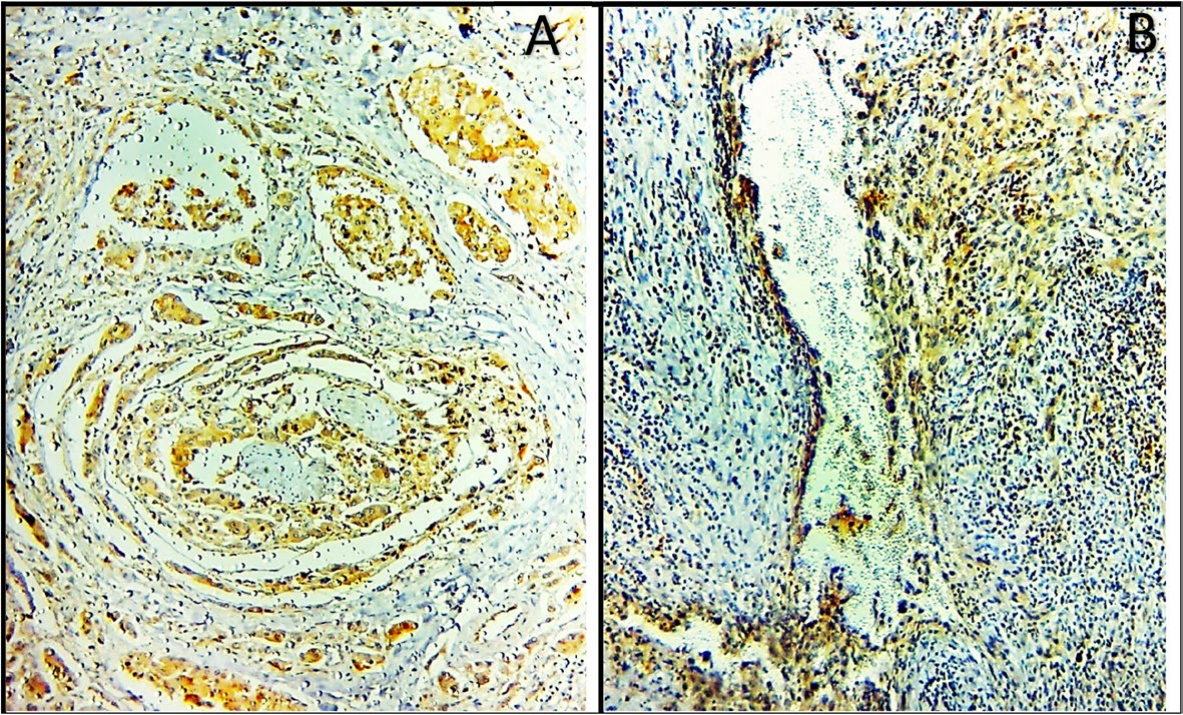
Positive CTSK expression in the cancerous cells that reveal perineural (**A**), and lymphovascular invasion (**B**) (× 400)

CTSK was not detected in the normal salivary gland tissue. In SGCs, CTSK was present mainly in carcinoma cells, but sometimes present in stromal cells. CTSK-positive cells at the stroma were present surrounding the invasive front. we observed that metastatic SGC cells in lymph nodes were also positive for CTSK (Fig. 1). The vast majority of metastasis-free lymph nodes did not express CTSK. A higher percentage of the worked cases had positive CTSK expressions (82.2%), and only eight of the worked cases had negative expressions (17.8%). Weak expression was observed in eight cases (17.8%), six cases (13.3%) showed moderate expression and 23 cases (51.1%) had strong CTSK expression. Regarding patient gender, tumor site, and the incidence of death, Pearson Chi-square test revealed no statistically significant differences in CTSK expression among the different groups (*P* values were 0.799, 0.801, and 0.0.078 respectively). On the other hand, there were statistically significant differences in CTSK expression concerning the following variables; histologic grade of tumor (*P* = 0.000), histologic type of carcinoma (*P* = 0.000), size of the tumor (*P* = 0.000), the status of nodal involvement (*P* = 0.041), distant metastasis (*P* = 0.009), TNM clinical stage (*P* = 0.000), and the incidence of recurrence (*P* = 0.009).

**Fig. 2:**
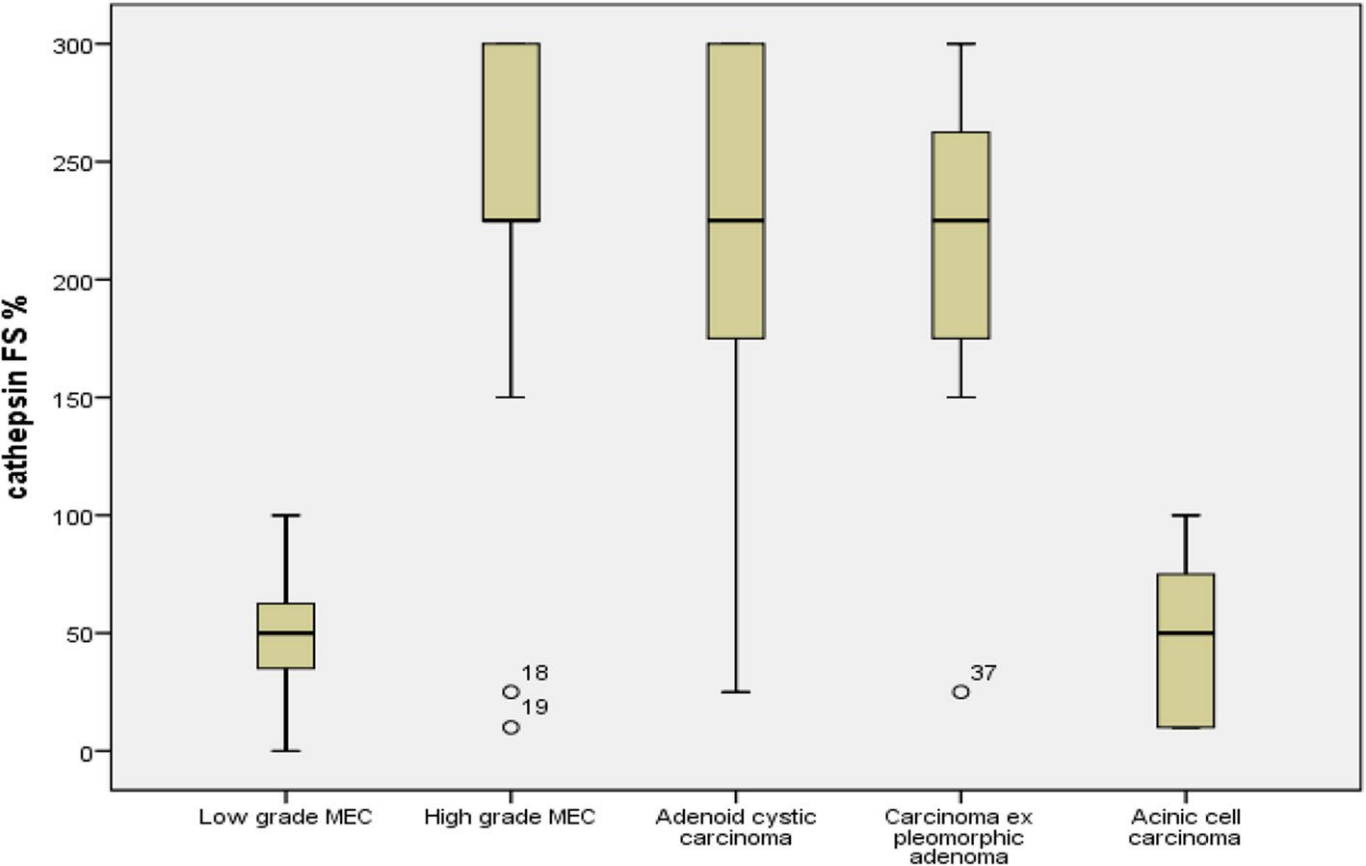
Cathepsin K immunohistochemical expression in the different histologic types of SGCs

**Fig. 3:**
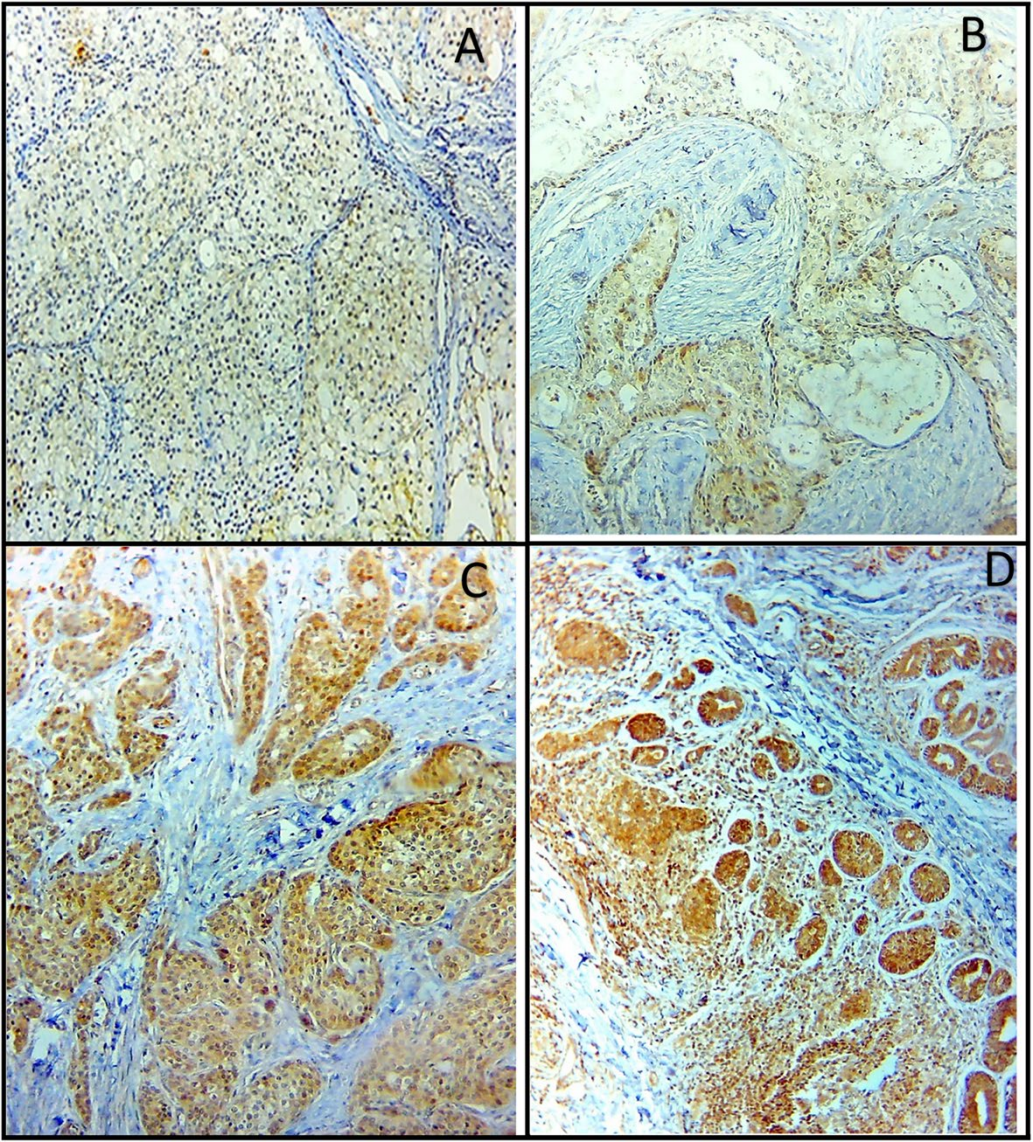
CTSK Immunohistochemical expression in SGCs; weak CTSK expression in (**A)** ACC and (**B)** low-grade MEC, strong CTSK expression in (**C)** high-grade MEC, and (**D)** AdCC (× 250)

**Fig. 4:**
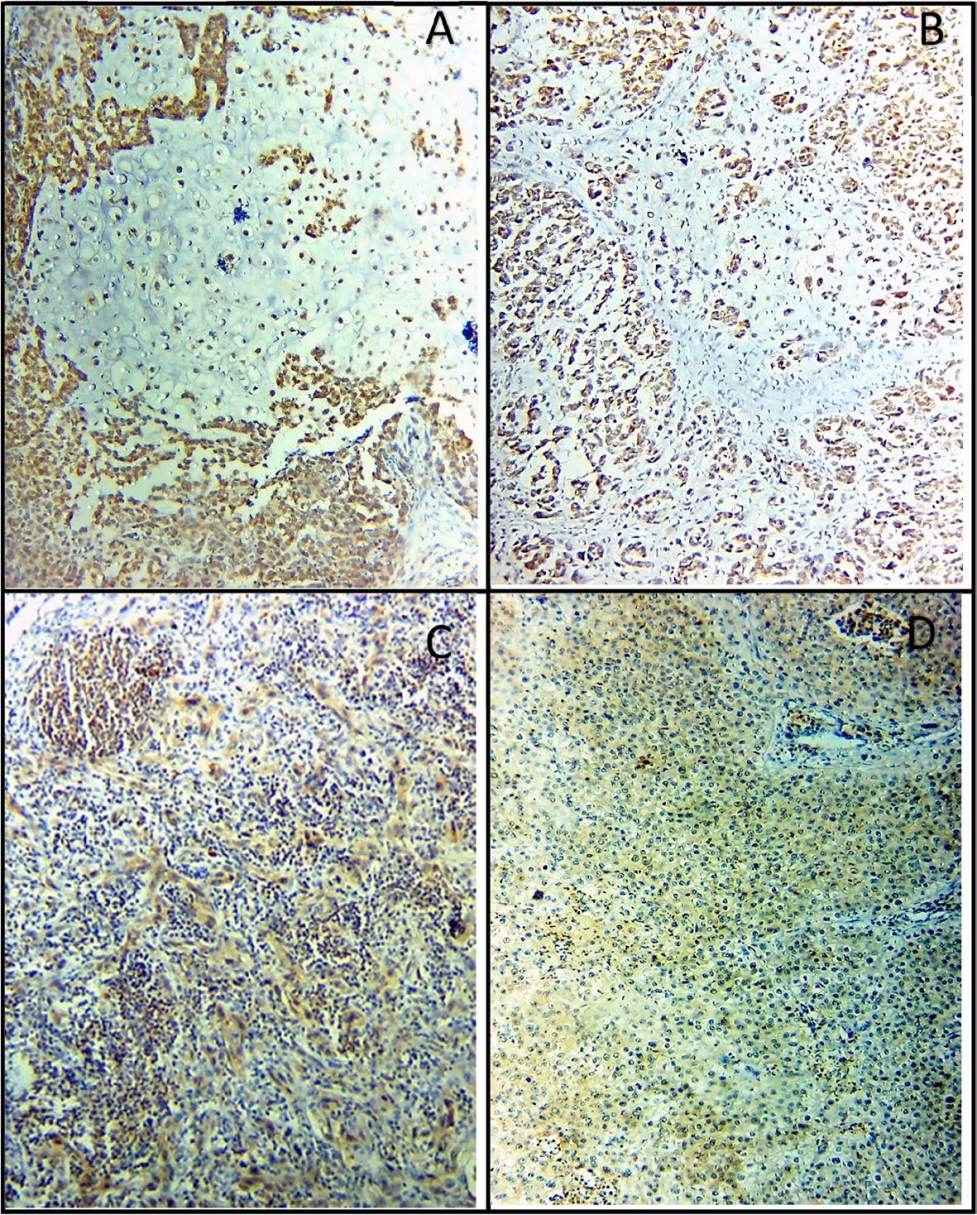
Strong CTSK IHC expression in CXPA (**A**, **B**), positive CTSK expression in the lymph nodes of (**C)** metastatic CXPA, and (**D)** high-grade MEC (× 250)

High-grade tumors revealed mainly strong (23 cases, 69.7%) and moderate (6 cases, 18.2%) CTSK expression. On contrary, low-grade carcinomas demonstrated weak (8 cases, 66.7%) and negative (4 cases, 33.3%) expression (Table 2). High-grade MEC mainly presented strong CTSK expression (10 cases, 76.9%), about two-thirds of AdCC presented strong expression of CTSK (8 cases, 66.7%) and five out of eight cases (62.5%) of CXPA had strong expression. Moderate CTSK expression was characteristically observed only in high-grade carcinomas; one case of high-grade MEC (7.7%), three cases of AdCC (25%), and two cases of CXPA (25%). Low-grade carcinomas mainly presented weak CTSK expression; five cases of low-grade MEC (71.4%) and three cases of ACC (60%). Negative CTSK expression was observed in two cases of ACC (40%), two cases of low-grade MEC (28.6%), two cases of high-grade MEC (15.4%), one case of AdCC (8.3%), and one case of CXPA (12.5%, Fig. 2, collected Figs. 3, 4).

**Table 2:** Cathepsin K immunohistochemical expression concerning the different histologic grades using Pearson chi-square test

Histologic grade	Cathepsin K		Total	Pearson Chi-Square Asymp. Sig. (2-sided)
	Negative or Weak expression	Moderate or Strong expression		
Low grade carcinomas % within Histologic grade	12	0	12	.000
	100.0%	0.0%	100.0%	
High grade carcinomas % within Histologic grade	4	29	33	
	12.1%	87.9%	100.0%	
Total % within Histologic grade	16	29	45	
	35.6%	64.4%	100.0%	

The mean difference is significant at the 0.05 level, Pearson Chi square test

The Chi-square test revealed a high statistically significant difference in CTSK IHC expression concerning the different sizes of tumors (*P* = 0.000, Table 3). large sized carcinomas (T3 and T4) showed mainly strong (19 cases, 73.1%) and moderate (4 cases, 15.4%) expression, while small-sized carcinomas (T1 and T2) showed negative (7 cases, 36.8%) and weak (6 cases, 31.6%) CTSK expression. Table 4 illustrates multiple comparisons among the varied sizes of tumors concerning CTSK expression utilizing One-way ANOVA post hoc test for multiple comparison. T1 tumors show a significant difference in CTSK expression in comparison with T2 (*p* = 0.011), T3 (*p* = 0.000), and T4 (*p* = 0.000) tumors. No difference in CTSK expression between T3 and T4 (*p* = 0.421) tumors.

**Table 3:** Cathepsin K immunohistochemical expression concerning the different tumor sizes using Pearson chi-square test

Tumor size	Cathepsin K		Total	Pearson Chi-Square Asymp. Sig. (2-sided)
	Negative or Weak expression	Moderate or Strong expression		
T1 + T2 % within tumor size	13	6	19	.000
	68.4%	31.6%	100.0%	
T3 + T4 % within tumor size	3	23	26	
	11.5%	88.5%	100.0%	
Total % within tumor size	16	29	45	
	35.6%	64.4%	100.0%	

The mean difference is significant at the 0.05 level, Pearson Chi square test

**Table 4:** CTSK expression concerning the different sizes of tumor using One-way ANOVA Post hoc test for multiple comparisons

(I) Tumor Size	(J) Tumor Size	Mean Difference (I-J)	Std. Error	Sig	95% Confidence Interval	
					Lower Bound	Upper Bound
**T1**	**T2**	-1.155^a^	.436	.011	-2.03	-.27
	**T3**	-2.042^a^	.411	.000	-2.87	-1.21
	**T4**	-2.349^a^	.462	.000	-3.28	-1.42
**T2**	**T1**	1.155^a^	.436	.011	.27	2.03
	**T3**	-.887^a^	.345	.014	-1.58	-.19
	**T4**	-1.194^a^	.404	.005	-2.01	-.38
**T3**	**T1**	2.042^a^	.411	.000	1.21	2.87
	**T2**	.887^a^	.345	.014	.19	1.58
	**T4**	-.307	.378	.421	-1.07	.46
**T4**	**T1**	2.349^a^	.462	.000	1.42	3.28
	**T2**	1.194^a^	.404	.005	.38	2.01
	**T3**	.307	.378	.421	-.46	1.07

^a^ The mean difference is significant at the 0.05 level

Positive nodal metastasis was reported in 26 of the studied cases (57.7%). About two third of these cases (17 cases, 65.4%) presented strong CTSK expression. Distant metastasis was reported in 17 of the worked cases (37.7%). Strong CTSK expression was observed in 15 of these cases (88.2%). Pearson chi-square test revealed high statistically significant differences in CTSK expression regarding the status of nodal and distant metastasis (*p* values were 0.041, 0.009 respectively, Tables 5, 6).

**Table 5:** Cathepsin K immunohistochemical expression concerning the status of nodal involvement using Pearson chi-square test

Nodal involvement	Cathepsin K		Total	Pearson Chi-Square Asymp. Sig. (2-sided)
	negative or weak expression	moderate or strong expression		
positive nodal involvement % within Nodal involvement	6	20	26	.041
	23.1%	76.9%	100.0%	
negative nodal involvement % within Nodal involvement	10	9	19	
	52.6%	47.4%	100.0%	
Total % within Nodal involvement	16	29	45	
	35.6%	64.4%	100.0%	

The mean difference is significant at the 0.05 level, Pearson Chi square test

**Table 6:** Cathepsin K immunohistochemical expression concerning the incidence of distant metastasis using Pearson chi-square test

Incidence of metastasis	Cathepsin K		Total	Pearson Chi-Square Asymp. Sig. (2-sided)
	negative or weak expression	moderate or strong expression		
Present % within metastasis	2	15	17	.009
	11.8%	88.2%	100.0%	
Absent % within metastasis	14	14	28	
	50.0%	50.0%	100.0%	
Total % within metastasis	16	29	45	
	35.6%	64.4%	100.0%	

The mean difference is significant at the 0.05 level, Pearson Chi square test

CTSK expression was significantly varied among the different TNM clinical stages (Pearson chi- square test, *P* = 0.000, Table 7). Table 8 presents multiple comparisons between the four TNM stages according to CTSK expression. Cases of stage I demonstrated a significant difference in CTSK expression when compared with cases in the other stages; II (*P* = 0.044), III (*P* = 0.001), and IV (*P* = 0.000). No statistically significant difference was present between stage II and stage III cases (*P* = 0.281). Stage IV cases had a statistically significant difference in CTSK expression when compared with the other stages: I (*P* = 0.000), II (*P* = 0.009), III (*P* = 0.047).

**Table 7:** Cathepsin K immunohistochemical expression concerning the different TNM clinical stages using Pearson chi-square test

TNM clinical stage	Cathepsin K		Total	Pearson Chi-Square Asymp. Sig. (2-sided)
	negative or weak expression	moderate or strong expression		
stage I + II % within TNM clinical stage	10	3	13	.000
	76.9%	23.1%	100.0%	
stage III + IV % within TNM clinical stage	6	26	32	
	18.8%	81.3%	100.0%	
Total % within TNM clinical stage	16	29	45	
	35.6%	64.4%	100.0%	

The mean difference is significant at the 0.05 level, Pearson Chi square test

**Table 8:** CTSK expression concerning the different TNM clinical stages using One-way ANOVA Post hoc test for multiple comparisons

(I) TNM clinical stage	(J) TNM clinical stage	Mean Difference (I-J)	Std. Error	Sig	95% Confidence Interval	
					Lower Bound	Upper Bound
**stage I**	**stage II**	-1.071^a^	.516	.044	-2.11	-.03
	**stage III**	-1.571^a^	.435	.001	-2.45	-.69
	**stage IV**	-2.256^a^	.410	.000	-3.08	-1.43
**stage II**	**stage I**	1.071^a^	.516	.044	.03	2.11
	**stage III**	-.500	.458	.281	-1.43	.43
	**stage IV**	-1.184^a^	.435	.009	-2.06	-.31
**stage III**	**stage I**	1.571^a^	.435	.001	.69	2.45
	**stage II**	.500	.458	.281	-.43	1.43
	**stage IV**	-.684^a^	.334	.047	-1.36	-.01
**stage IV**	**stage I**	2.256^a^	.410	.000	1.43	3.08
	**stage II**	1.184^a^	.435	.009	.31	2.06
	**stage III**	.684^a^	.334	.047	.01	1.36

^a^ The mean difference is significant at the 0.05 level

During the periodic follow-up events following the treatment, recurrence was reported in 17 of the worked cases (37.7%). Strong CTSK expression was significantly observed in the majority of recurrent cases (14 cases, 82.4%). Pearson chi-square test revealed a high statistically significant difference in CTSK expression regarding the incidence of recurrence (*p* = 0.009, Table 9).

**Table 9:** Cathepsin K immunohistochemical expression concerning the incidence of recurrence using Pearson chi-square test

Incidence of recurrence	Cathepsin K		Total	Pearson Chi-Square Asymp. Sig. (2-sided)
	negative or weak expression	moderate or strong expression		
Present % within recurrence	2	15	17	.009
	11.8%	88.2%	100.0%	
Absent % within recurrence	14	14	28	
	50.0%	50.0%	100.0%	
Total % within recurrence	16	29	45	
	35.6%	64.4%	100.0%	

The mean difference is significant at the 0.05 level, Pearson Chi square test

Death was reported in five of the worked cases. All these cases demonstrated strong CTSK expression. Pearson chi-square test revealed no statistically significant difference in CTSK expression concerning the incidence of death (*P* = 0.078).

### Disease Free survival (DFS) & 3-years Overall Survival (OS)

**Fig. 5:**
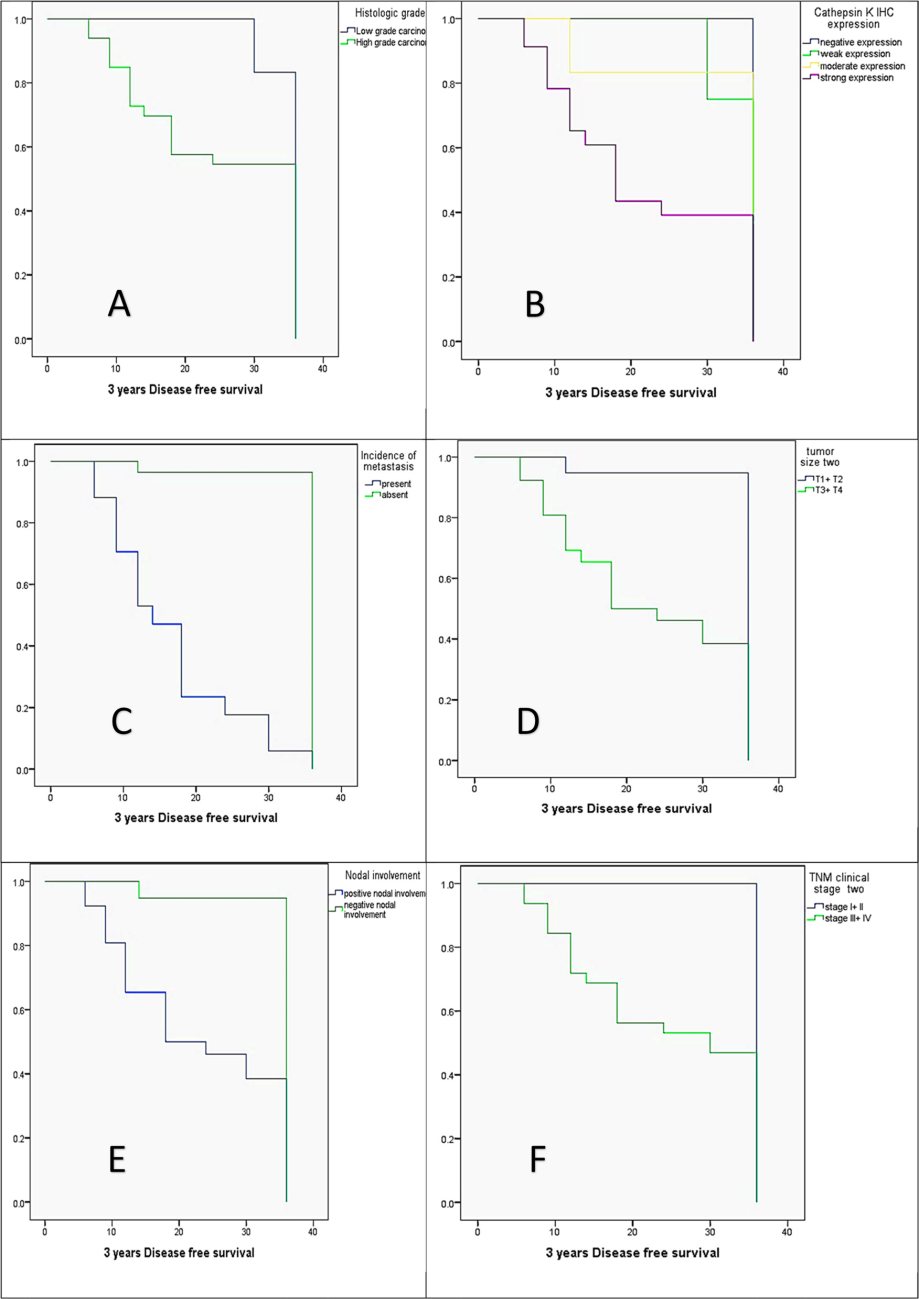
The Kaplan Meier survival plots demonstrate the 3-year of DFS significantly reduced in (**A)** the high histologic grade, (**B)** the strong CTSK expression, (**C)** the presence of metastasis, (**D)** the large-sized tumors (T3 + T4), (**E)** the positive nodal involvement, and (**F)** the advanced TNM clinical stage (III + IV)

Patients` DFS and 3-years OS were analyzed concerning the different clinicopathologic variables using the Kaplan Meier method, log-rank test, and the Cox regression model. Univariate analysis using Kaplan Meier method revealed that DFS was significantly reduced in high-grade carcinomas (25.6 months) versus low-grade carcinomas (35 months, *P* = 0.05), cases that had strong CTSK expression (22 months) versus negative (36 months), weak (34 months), and moderate (32 months) CTSK expressions (*P* = 0.006), positive distant metastasis (16.5 months) versus negative distant metastasis (35 months, *P* = 0.000), large sized carcinomas (T3 + T4; 23.2 months) versus small sized carcinomas ( T1 + T2; 34.7 months, *P* = 0.000), positive nodal involvement (23.19 months) versus negative nodal involvement( 34.8 months, *P* = 0.000), advanced TNM stage (stage III + IV; 24.9 months) versus stage I + II cases (36 months, *P* = 0.002). In contrary DFS had no statistically significant difference concerning gender and tumor site variables (*p* > 0.05, Fig. 5).

Multivariate analysis using the cox regression model found that distant metastasis is the independent predictor for DFS (Table 10).

**Table 10:** The Cox regression model illustrates the independent predictor(s) of DFS

**Variables**	B	SE	Wald	df	Sig	Exp(B)	95.0% CI for Exp(B)	
							Lower	Upper
**Histologic grade**	-.046	.720	.004	1	.950	.956	.233	3.919
**CTSK**			.118	3	.990			
**CTSK (1)**	-.025	.633	.002	1	.968	.975	.282	3.369
**CTSK (2)**	-.153	.845	.033	1	.856	.858	.164	4.498
**CTSK (3)**	.145	.577	.063	1	.801	1.156	.373	3.582
**Metastasis**	2.060	.615	11.228	1	.001	7.843	2.351	26.164
**Tumor size**	.369	.679	.296	1	.586	1.447	.382	5.474
**Nodal status**	-.104	.628	.027	1	.868	.901	.263	3.087
**TNM stage**	-.277	.908	.093	1	.760	.758	.128	4.488

**Fig. 6:**
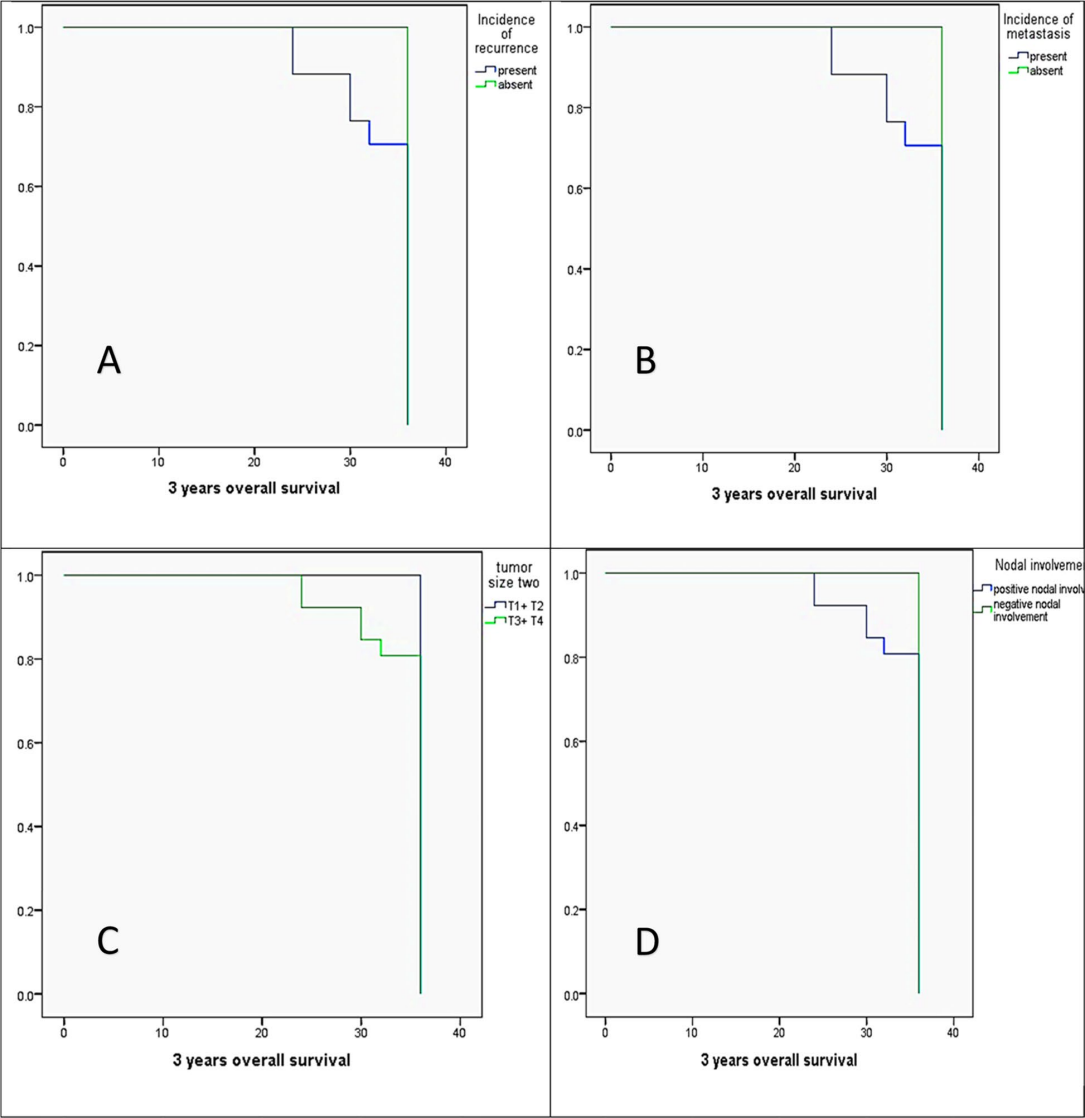
The Kaplan Meier survival plots demonstrate the 3-year of OS significantly reduced in (**A)** the presence of recurrence, (**B)** the presence of distant metastasis, (**C)** the large-sized tumors (T3 + T4), and (**D)** the positive nodal involvement

Univariate analysis of the 3 years OS concerning different clinicopathologic variables using the Kaplan Meier method revealed that 3 years OS was significantly reduced in cases that had recurrence during follow-up (28 months) versus recurrence-free cases (36 months, *P* = 0.002), positive distant metastasis (33.6 months) versus negative distant metastasis (36 months, *P* = 0.002), positive nodal involvement (34.4 months) versus negative nodal involvement (36 months, *P* = 0.046), large sized carcinomas (T3 + T4, 34.4 months) versus small sized carcinomas (T1 + T2, 36 months, *P* = 0.046). In contrast 3 years OS had no statistically significant differences concerning CTSK expression (*P* = 0.154), tumor grade (*P* = 0.162), tumor histologic type (*P* = 0.418), TNM stage (*P* = 0.140), tumor site (*P* = 0.25), and gender variables (*P* = 0.059, Fig. 6).

Multivariate analysis using the Cox regression model found that none of the studied variables could predict 3 years of OS (Table 11).

**Table 11:** The Cox regression model illustrates the independent predictor(s) of OS

**Variables**	B	SE	Wald	df	Sig	Exp(B)
Recurrence	.160	.680	.055	1	.814	1.174
Metastasis	.155	.750	.043	1	.836	1.168
Tumor size	-.005	.410	.000	1	.991	.995
Nodal involvement	.036	.366	.010	1	.921	1.037

## Discussion

The diagnosis of cancer patients is usually an event at the advanced stage of cancer owing to the deficient use of accurate biomarkers in clinical settings that reflects the progression of cancer. For that reason, specific biomarkers are critically required to diagnose tumors in clinical settings [30]. The biomarkers are useful in determining high-risk individuals, the aggressiveness of the tumor, prediction of metastasis, monitoring tumor progression, developing customized therapies for patients with different cancers, and assessing the efficacy and outcomes of disease treatment.

CTSK has a complex role in cancer. It participates in cancer diagnosis due to its physiological role in bone remodeling and resorption, degradation of extracellular matrix, angiogenesis, and progenitor cell mobilization. In preclinical or clinical studies, high CTSK expression was detected in the serum and tissues of cancer patients. The majority of cancers showed bone metastases at the advanced stage that cause massive damage to patients [24]. Although there are several mechanisms of tumor growth, metastasis, and cancer cell invasion related to CTSK, its expression in primary tumors including salivary gland carcinomas has not been thoroughly investigated [23, 24]. In our study, we studied the possible role of CTSK in SGCs and searched for the correlations between its expression and the different clinicopathological variables.

In the current study, positive CTSK expression is present in 82.2% of the worked cases, and 87.87% of high-grade SGCs present moderate and strong CTSK expression. In contrast, low-grade SGCs present negative (4 cases, 33.3%) and weak expression (8 cases, 66.7%). There were statistically significant differences in CTSK immunoexpression concerning the following different variables; tumor type, tumor histologic grade, tumor size, the status of nodal involvement, metastasis, TNM clinical stage, and the incidence of recurrence (*p* < 0.05). Also, there were no statistically significant differences in CTSK expression concerning patient`s gender, tumor site, and the incidence of death variables using Pearson chi-square and one-way ANOVA tests.

CTSK significantly revealed strong expression in large-sized carcinomas (19 cases, 73.1%), while small-sized carcinomas showed negative (7 cases, 36.8%) and weak (6 cases, 31.6%) expression. Moreover, strong CTSK expression was significantly reported in carcinomas that had positive nodal and distant metastasis. CTSK facilitates the degradation of the ECM, enabling the migration and proliferation of cancer cells that explains the strong expression which was observed in carcinomas of aggressive behavior. Many studies agreed with our findings. Sivaranjini Y et al., (2012) [31] observed intense cathepsin D (CTSD) expression in high-grade SGCs. CTSD was significantly expressed in AdCC than in polymorphous low-grade adenocarcinoma (PLGA) [31]. They recommended the use of CTSD as a marker of invasive potential and aggressive behavior.

Moreover, Zhang et al. (2018), noted high CTSD expression was related to unfavorable clinicopathologic characteristics such as perineural invasion, advanced clinical stage, reduced survival, and distant metastasis. AdCC cases revealed high CTSD expression than normal salivary glandular tissue. CTSD Si RNA treatment makes morphological alteration of cancer cells from mesenchymal-like cells to epithelioid cells. CTSD accelerates the migration and invasion of cells via ultrastructural modification and pseudopodia formation in SACC- LM cells, in addition to its proteolytic activity in the tumor microenvironment [32].

CTSK overexpression is associated with cancer metastatic disease with a potential prognostic value [24, 33]. CTSK promotes metastasis of gastric cancer cells by potentiating remodeling of ECM through activation of MMP5 [34, 35]. Studies on colorectal cancer also support our finding, invasion and metastasis of colorectal cancer cells promoted by CTSK that stimulates the release of cytokines such as IL10 and IL17 through activation of the mTOR pathway. Metastasis is the main cause of death in colorectal cancer patients. High CTSK expression was discovered as a novel marker for metastasis. Its expression was associated with poor prognostic outcomes. These findings present the predictive role of CTSK in colorectal cancer and the validity to use it as a therapeutic target [36, 37].

On the other hand, studies on melanoma confirmed the vital role of CTSK in the acquisition of aggressive behavior by melanoma cells. CTSK not only promotes metastasis but also could predict it. CTSK is an independent predictor of metastasis. Melanomas release CTSK and MMP to cut collagen in the intima of lymph and blood vessels. CTSK promotes motility and migration of melanocytes through the dermis and realizes distant metastasis [38, 39]. Breast cancer studies also acknowledged that CTSK activates pro MMP9 to produce MMP9 that potentiates migration of breast cancer cells to establish distant metastasis [40–44]. Strong CTSK expression was noted in human breast cancers with primary and developing bone metastasis. Breast cancer studies on CTSD were recognized as a marker of poor prognosis [35, 45]. Additionally, bone tumor studies acknowledged elevated CTSD expression promotes bone metastasis and bone tumor progression [46, 47]. Bone metastasis occurred due to imbalanced resorption and formation of bone. Tumor cells inhibit the formation of osteoblasts [48]. Multiple myelomas and tumor cells initiate bone resorption by secretion of factors that activates the RANK/RANKL signaling pathway [49, 50].

Inhibition of CTSK can significantly inhibit the mTOR signaling pathway [51]. The mTOR pathway has a vital role in the maintenance of cell growth, proliferation, motility, and survival that is involved in the development of a variety of cancers [52, 53]. Many studies on kidney cancer recognized that CTSK’s high expression demonstrates the progression of cancer [54–58]. The activated mTOR signaling pathway was related to the development of renal cell carcinoma [59, 60].

Many researchers assessed the potential role of CTSK in lung cancer. CTSK was expressed in non-small cell lung cancer (NSCLC) as adenocarcinoma, adenosquamous carcinoma, squamous cell carcinoma (SCC), and large cell carcinoma, but rarely studied in small cell lung carcinoma (SCLC). Contradictory to our finding regarding the negative expression of CTSK in normal salivary gland tissue, the authors reported positive CTSK expression in normal salivary gland tissue. Additionally, CTSK is acknowledged as a potential biomarker for pulmonary perivascular epithelioid tumors due to its diffuse and strong expression [61]. Wang and his coworkers reported elevated CTSK levels at tumor-associated macrophages in NSCLC [62]. In 2020, Yang et al. also recognized a significant elevation of CTSK in A549 cells of NSCLC. CTSK overexpression was associated with increased proliferation, migration, and invasion of cells by activation of the mTOR pathway [63]. Moreover, inhibition of CTSK inhibits cell proliferation and distant metastasis of ovarian cancer cells by suppressing epithelial-mesenchymal transition [64].

Contradictory to our findings, a study carried out on tongue SCC revealed that CTSK expression had no correlations to gender and age of patients similar to ours, but they presented contradictory results regarding the grade of carcinoma, the clinical stage, and the nodal status. Moreover, they also observed a correlation between the diminished CTSK expression in the tumor microenvironment (TME) and the increased overall recurrence [65].

In the present study, the higher incidence of distant metastasis was significantly encountered in SGCs of high grade as follows; ((7 cases, 53.8%) of high-grade MECs, (5 cases, 41.7%) of AdCC, (3 cases, 37.5%) of CXPA). Distant metastasis also was related to strong CTSK expression (15 cases, 65.2% of carcinomas demonstrated strong CTSK expression), presence of recurrence during follow-up (16 cases, 94.1% of cases that had recurrence), large-sized tumors (17 cases, 65.4% of T3 and T4 tumors), positive nodal involvement (16 cases, 61.5% of nodal positive cases), all the dead cases (5 cases, 100% of deaths), advanced TNM clinical stage (17 cases, 53.1% of stage III and IV cases) (*P* values were < 0.05).

Multivariate analysis using the cox regression model found that distant metastasis was the independent predictor for DFS. On the other hand, there was no statistically significant relationship between the incidence of metastasis to the tumor site and the gender of patients (*P* > 0.05). In the same line with our findings, multivariate analysis of other studies revealed intermediate and high-risk histology, advanced T classification, and neck node disease were independently associated with the development of distant metastasis. High-grade histologies, advanced T classification, and neck disease are considered risk factors for distant metastasis [66–68]. Moreover, these studies did not find a relationship between the male sex and the incidence of distant metastasis [66–68].

Opposite to our finding, one study found about a 1.4-fold higher risk of distant metastasis in males [44].

## Conclusions

Diagnosis of distant metastasis in malignant tumors is often a late and difficult event due to the lack of specific tumor biomarkers. As we discussed in advance several studies have reported a high level of CTSK expression associated with metastasis of cancer cells. Also, CTSK inhibition reduces the progression of osteolytic lesions, indicating the significance of CTSK as a tumor biomarker [69–71]. Best to our Knowledge, our study is the first study that present the role of CTSK in malignant salivary gland tumors. Strong CTSK expression was related to poor clinicopathological parameters such as (high-grade carcinomas, large infiltrating carcinomas, presence of nodal involvement, presence of distant metastasis, carcinomas of advanced TNM clinical stage, presence of recurrence, and reduced DFS). The great role of CTSK in cancer progression through triggering many signaling pathways indicates its utility as a therapeutic target for cancer treatment [25, 72, 73]. Therefore, the level of CTSK in cancer tissue is considered an effective index for predicting the severity and prognosis of cancers (Table 12).

**Table 12:** Previous studies present the role of Cathepsin B and Cathepsin D in salivary gland tumors

Salivary gland neoplasm type	casuistry	antibodies	Analysis method	Main results	Statistically significant
Salivary Adenoid cystic carcinoma (SACC)	Role of CTSB in cancer metastasis by altering extracellular matrix (ECM) remodeling and facilitating invasion [74]	anti-CTSB	Cell culture and transfection. (SACC-83 cells) Reverse transcription-quantitative polymerase chain reaction (RT-qPCR), western blot analysis, immunofluorescence staining, migration and invasion assays, and 3D spheroid culture Immunohistochemistry (IHC) [74]	CTSB overexpression in the invasive front of SACC compared to the tumor center CTSB was only expressed in leader cells CTSB expression associated with a poor prognosis of patients with SACC 3D spheroid invasion assay provides evidence that CTSB may define leader cells in SACC and is required for collective cell invasion as a potential key regulator of ECM remodeling [74]	The increased CTSB expression at the invasion border was closely associated with a poor pathological type, nerve invasion, advanced clinical tumor-node-metastasis stage, recurrence and metastasis High CTSB level was associated with reduced overall survival [74]
SACC	Assess the functional role of CTSD in perineural invasion (PNI) of SACC [32]	Anti CTSD	QRT-PCR, immunofluorescence and western blot analysis were used to examine the levels of CTSD mRNA and protein in SACC-LM cell line [32]	The ability of migration, invasion, and PNI could be inhibited significantly by siRNA mediated CTSD silence (*p* < 0.01). Furthermore, siRNA-mediated CTSD silence inhibited cytoskeletal organization and pseudo foot formation in SACC-LM cells [32]	High CTSD in solid pattern than that with tubular pattern of SACC CTSD correlated with clinic stage and distant metastasis High CTSD expression was significantly shorter OS [32]
	Intense expression of CTSD in high grade carcinomas may be a marker for invasive potential and aggressive behavior [31]	anti- CTSD antibody	Immunohistochemistry [31]		Intense CTSD expression in adenoid cystic carcinoma (ACC) and mucoepidermoid carcinoma (MEC) as compared to polymorphus low grade adenocarcinoma (PLGA) [31]
Mucoepidermoid carcinoma (MEC), adenoid cystic carcinoma (ACC), Warthin’s tumor (WT) and pleomorphic adenoma (PA) [75]	CTSD is a useful marker for the aggressive biologic behavior as well as invasive potential of salivary gland neoplasms [75]	anti- CTSD antibody	Immunohistochemistry	Significant differential CTSD expression between benign and malignant salivary gland tumors [75]	Malignant salivary gland tumors showed significant high CTSD expression when compared to the benign tumors [75]

## Data Availability

The data used during the current study are available from the corresponding author on a reasonable request.

## Abbreviations

SGCs: Salivary gland carcinomas
CTSK: Cathepsin K
AdCC: Adenoid cystic carcinoma
CXPA: Carcinoma ex pleomorphic adenoma
MEC: Mucoepidermoid carcinoma
ACC: Acinic cell carcinoma
IHC: Immunohistochemistry
